# The epidemiology of undernutrition and its determinants in children under five years in Ghana

**DOI:** 10.1371/journal.pone.0219665

**Published:** 2019-07-31

**Authors:** Michael Boah, Fusta Azupogo, Daniel Adjei Amporfro, Linda Anutua Abada

**Affiliations:** 1 Ghana Health Service, Bolgatanga, Upper East Region, Ghana; 2 Department of Family and Consumer Sciences, Faculty of Agriculture, University for Development Studies, Tamale, Ghana; 3 Department of Social Medicine and Health Service Management, Harbin Medical University, Harbin, China; Kansas State University, UNITED STATES

## Abstract

**Background:**

Understanding the burden and contextual risk factors is critical for developing appropriate interventions to control undernutrition.

**Methods:**

This study used data from the 2014 Ghana Demographic and Health Survey to estimate the prevalence of underweight, stunting, and wasting. Single multiple logistic regressions were used to identify the factors associated with underweight, wasting and stunting. The study involved 2720 children aged 0–59 months old and mother pairs. All analyses were done in STATA/IC version 15.0. Statistical significance was set at p<0.05.

**Results:**

The prevalence of underweight, wasting and stunting were 10.4%, 5.3%, and 18.4% respectively. The age of the child was associated with underweight, wasting and stunting, whereas the sex was associated with wasting and stunting. Normal or overweight/obese maternal body mass index category, high woman’s autonomy and middle-class wealth index were associated with a lower odds of undernutrition. The factors that were associated with a higher odds of child undernutrition included: low birth weight (<2.5 kg), minimum dietary diversity score (MDDS), a higher (≥4^th^) birth order number of child, primary educational level of husband/partner and domicile in the northern region of Ghana.

**Conclusion:**

There is still a high burden of child undernutrition in Ghana. The age, sex, birth weight, birth order and the MDDS of the child were the immediate factors associated with child undernutrition. The intermediate factors that were associated with child undernutrition were mainly maternal related factors and included maternal nutritional status and autonomy. Distal level factors which were associated with a higher odds of child undernutrition were the wealth index of the household, paternal educational status and region of residence. We recommend that interventions and policies for undernutrition should address socioeconomic inequalities at the community level while factoring in women empowerment programmes.

## Introduction

Malnutrition is a major cause of death among children under five years of age. An estimated 13.6 million children die annually from undernutrition globally [1]. Mortality in children from undernutrition is highest in developing countries [2]. At the global level, an estimated 151 million (22.2%) children under-five years of age were stunted in 2016 [3]. An additional 51 million (7.5%) were at risk of wasting in the same year. Stunting and wasting rates in Africa are above global estimates, albeit inter-country variations. In 2016, stunting affected an estimated 39% of children under-five years while wasting threatened the lives of an estimated 27% [4].

The causes of malnutrition are multifaceted and include diseases, inadequate diet, environmental, and socioeconomic factors [5]. The age of the child, gender, birth weight, child’s vaccination status, birth spacing, birth order, maternal education, mother’s body mass index (BMI), antenatal care (ANC) use by mother, household wealth index, improved water, hygiene and sanitation, family structure, and family size have been identified as some of the determinants of children’s nutritional status in sub-Saharan Africa [6–11].

The consequences of poor nutrition during infancy and childhood have been well documented and include impaired growth, poor cognitive and social development, poor school performance, increased risk of morbidity and mortality and reduced productivity later in life [2,12–14]. Stunting is linked to poor environmental conditions and repeated exposure to adverse economic conditions that result from poor nutrition during pregnancy and early childhood [15]. Wasting is a life-threatening result of insufficient food intake and/or disease; it is a measure of acute malnutrition [16]. Nevertheless, the nutritional status of children can serve as an indicator for measuring the health and well-being of populations; because early childhood health indicators are sensitive to food security situations, environmental, economic and policy changes [17–19]. Thus, they reflect the living conditions to which the child is “exposed” to.

The 2014 Ghana Demographic and Health Survey (GDHS) report shows a decline in neonatal, infant and young child mortality over the past two decades; similarly, malnutrition rates have declined over the same period but remain alarming [20]. Underweight is falling too slowly while stunting and wasting still impact on the lives of many more children under-five years of age. As a result, both small and large-scale studies have assessed the determinants of childhood undernutrition in Ghana [21–25]. However, these studies are either not generalizable to other parts of the country due to their small sample sizes and location of study [24,25], or they focused on one aspect of undernutrition [21–23].

This study sought to estimate the prevalence of underweight, wasting and stunting, and explore their determinants among children under five years old in Ghana while recognizing the complex hierarchical relationship of these determinants. The findings would be useful in the formulation of policies in Ghana to tackle undernutrition and contribute to the literature by providing evidence on the determinants of the commonly used nutrition profiles for defining undernutrition in children under five at the national level [15,19].

## Methods

### Conceptual framework

The conceptual framework for this study was founded on previous studies that have identified and described risk factors of malnutrition in children [5,9,23,26]. The framework used is based on the premise that distal factors may determine the nutritional status of children by acting directly or indirectly through some interrelated mediating factors except for age and gender of the child. Briefly, according to our framework, the immediate causes of childhood undernutrition include food, birth weight, the birth order number of the child and diseases. Infections and diarrhoea can decrease food intake and nutrient utilization resulting in poor nutrition, growth, and development of the child [27,28].

Also, the immediate causes of childhood undernutrition are rooted in problems at the household level. Maternal undernutrition during pregnancy can result in low birth weight at birth, which is associated with an increased risk of undernutrition in early childhood [29]. Large family sizes may lead to inadequate food intake, as do poor access to safe water and sanitation facilities lead to an increase in diseases, which in turn affects food intake and utilization [30]. Caregivers may seek care for their sick children when health care services are accessible and affordable.

Furthermore, each household level problem, in turn, has its correlated factors at the distal level. As a fact, some household behaviours are modelled by cultural and religious norms prevalent in the community [5]. Education, employment, household wealth and place of residence are indices of socioeconomic status and may reflect access to resources by the household [28]. A higher maternal educational level and household wealth index are associated with increased access to household dietary needs, health care services and better living conditions, which are inhibitors of childhood undernutrition [31,32].

**Fig 1:**
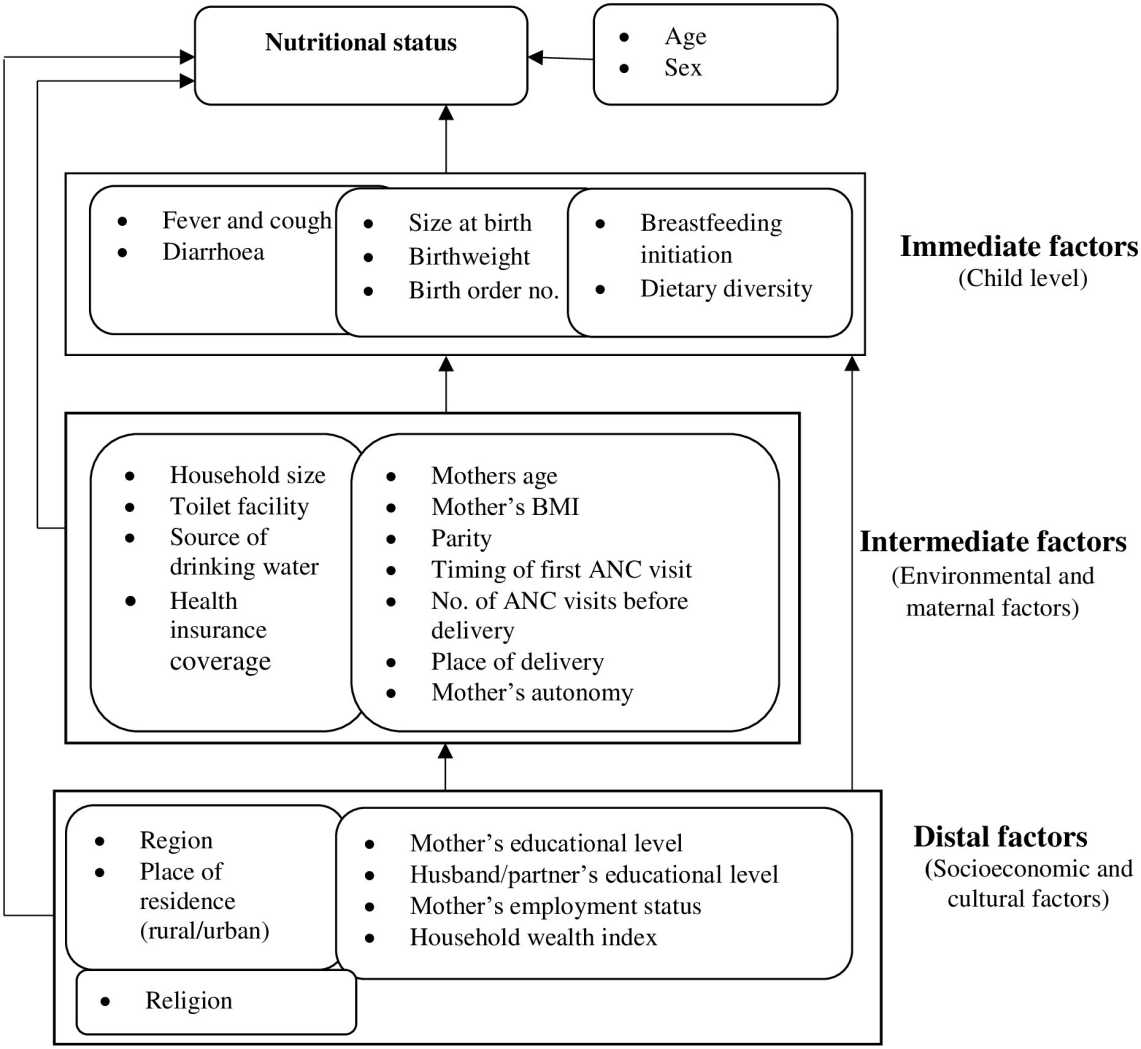
A conceptual framework of the determinants of child undernutrition (source: Adapted from Hein & Hoa, 2009; Mostafa, 2011; Nikoi & Anthamatten, 2014; United Nations Children’s Fund, 1991).

Our framework lays out the hierarchical relationship between the risk factors for childhood undernutrition that were examined in this study (Fig 1).

### Source of data

We used the child recode dataset of the 2014 GDHS. Approval for the use of the dataset was obtained from ICF international. The Demographic and Health Survey (DHS) is a nationally representative survey that provides coverage data at the population level on key health indicators including reproductive health, fertility, child health, and nutrition from which differences can be assessed by bio-demographic, socioeconomic and geographic characteristics after disaggregation. Details about the survey can be found in the DHS Methodology report [33].

The 2014 GDHS was carried out by the Ghana Statistical Service (GSS), Ghana Health Services (GHS), and the National Public Health Reference Laboratory (NPHRL) of the GHS. The survey employed a multistage and multi-sampling technique. Sampling units (clusters) were selected in the first stage. The second stage involved the systematic selection of 12, 831 households. Three different questionnaires were used to collect information on household characteristics, fertility, morbidity, mortality and child health. Eligible women for interview were all women aged 15–49 years who were either permanent residents or visitors who stayed in a selected household the night preceding the survey. Weight and height measurements were collected from eligible women and children 0–59 months. Children from selected households were measured irrespective of whether their mothers were interviewed. The sampling frame used was updated from the 2010 population and housing census (PHC). The response rate was 97% for the women’s questionnaire. Height and weight measurements were taken for 3,118 children 0–59 months. However, anthropometric information was available for 2,895 children in the dataset. We excluded children who were flagged for z-scores of nutritional status indices (n = 175), which led to the final sample of 2720 (weighted n = 2636) children under five years of age for analysis. Further details on the survey design and data collection process have been explained elsewhere [20]. Some variables were recategorized to produce enough sample for data analysis.

### Variables

#### Dependent variables

The three dependent variables in this study were underweight, wasting and stunting. Weight-for-age (WAZ), weight-for-height (WHZ) and height-for-age (HAZ) z-scores of less than -2 standard deviations (SD) from the median according to the 2006 child growth standards of the World Health Organization (WHO) were used to define underweight, wasting and stunting respectively [34].

The z-scores cut-off point was used to construct binary measures of underweight (WAZ < -2SD), wasting (WHZ < -2SD) and stunting (HAZ < -2SD). A dummy variable with a value of “1” was used in each case to identify children who were underweight, wasted or stunted and “0” for children who are not underweight, wasted or stunted.

#### Independent variables

Control variables: the age (in months) and sex of the child were considered as control variables. Age was categorized as 0–5; 6–11; 12–23; 24–35; and 36+ months of age.

The immediate factors (child level factors) included in the study were child’s birth weight; child’s birth order number among other living children; dietary diversity score (DDS); fever and cough episode in the last two weeks before the survey; and diarrhoea episode in last two weeks before the survey. Fever, cough and diarrhoea were considered measures for child’s health status. A DDS comprising 7 food groups was created for the children based on the available data. The food groups included grains, roots and tubers; legumes and nuts; dairy products (cheese, milk, and yoghurt); flesh foods (meat, fish, poultry); eggs; vitamin A rich fruits and vegetables; and other fruits and vegetables [35]. In the DDS, a score of ‘1’ else ‘0’ was assigned if the child consumed at least one food item from each of the food groups. The aggregated scores of the 7 food groups comprised the DDS which ranged from 0–7. The acceptable minimum DDS was the consumption of foods from at least four food groups.

The intermediate factors (household and maternal factors) included in the study were mother’s age; mother’s parity; mother’s Body-Mass-Index (BMI) categorized as thin (BMI< 18.5kg/m^2^), normal (BMI 18.5–24.9 kg/m^2^) and overweight/obese (BMI ≥25 kg/m^2^); the timing of the first ANC visit; the place used for delivery by the mother; health insurance coverage; woman’s autonomy; household size; type of toilet facility; and source of drinking water. Antenatal care use in Ghana is almost universal [20]; hence, the timing of the first ANC visit and the place of delivery were used as measures of mother’s health-seeking behaviour during pregnancy and for childbirth. The woman’s autonomy was measured by her involvement in household decision making, attitude towards wife beating and property ownership. A cumulative autonomy index score was created from the summation of individual scores (see supporting material S1 Table). Tertiles of woman’s autonomy was constructed from the final autonomy index score to provide a measure for woman’s autonomy. This method has been used in other studies [36–38]. The categorization of the type of toilet facility and source of drinking water was guided by the World Health Organization & United Nations Children’s Fund definitions [39]. The definition of improved household toilet facility was adapted to take into consideration the housing system in some parts of Ghana [40–42]. Therefore, the use of ‘Improved household toilet’ in this study defined all households with access to improved toilet facilities, including those shared with other household members.

The distal factors (socioeconomic and cultural factors) considered were the administrative region, place of residence (rural or urban), mother’s educational level, husband/partner’s educational level, mother’s employment status, household wealth index and religion of the mother. Mother and husband/partner’s educational level was measured by three dummy variables; no formal education, primary, and secondary or higher. The wealth index is a composite measure of a household's cumulative living standard and was calculated in the GDHS by using easy-to-collect data on a household’s ownership of selected assets, such as televisions and bicycles; materials used for housing construction; and types of water access and sanitation facilities. Household wealth quintiles ranging from the poorest to the richest were used as a measure of the wealth index.

### Data analysis

It was presumed that the independent variables would exhibit different patterns of relationship across and within hierarchical levels of each of the dependent variables. Therefore, multiple single logistic regressions were preferred over multivariate regression models in identifying the determinants of underweight, wasting and stunting [43]. Besides, the “svy” command prefix used to adjust for the survey design used by the DHS program can be used with single logistic regression models and not multivariate regression models to produce robust coefficients, standard errors and confidence intervals that are representative. The model fitting process in this study involved three stages.

Firstly, the independent association of each of the distal factors with each of the forms of undernutrition in the absence of the intermediate and immediate factors was assessed (model 1). Secondly, distal factors were fitted with the intermediate factors to assess the association between the intermediate factors and undernutrition adjusting for the confounding effects of distal factors (model 2). Finally, the distal factors and intermediate factors were fitted with immediate factors; this produced the “best fit” independent association between the immediate factors and undernutrition while adjusting for the confounding effects of the distal and intermediate factors and the independent relationship between distal factors and undernutrition (model 3). The age and sex of the child were considered as control variables and maintained in each of the models. The model fitting process was guided by Victora, Huttly, Fuchs, & Olinto [44].

To avoid an excessive number of parameters and unstable estimates in subsequent models, only variables with a p-value <0.1 were retained in subsequent models [45]. We entered pairwise interaction terms in order to explore potential nonlinearities, but none of these interactions was statistically significant in the final models. Prevalence estimates with their corresponding confidence intervals (CI) were calculated for the dependent variables, and the Chi-square (χ^2^) test was used to assess significant differences between the groups. Adjusted odds ratios (AOR) with their corresponding 95% CIs were reported for risk factors. All data analyses were done using STATA/IC version 15.0 for Windows (StataCorp LLC, College Station, Texas USA). The ‘svyset’ and ‘svy’ command prefix, as well as weights, were used to adjust for the complex study design used by the DHS program.

### Ethics

This study did not need any ethical clearance because it is a secondary analysis of data from the 2014 GDHS. The dataset used for the analyses did not contain personal identifiers to respondents or households; the DHS Program protects the privacy of respondents and household members in the surveys. The DHS survey procedures were approved by the Institutional Review Board of ICF Macro International (Calverton, Maryland USA) and the Ethics Review Committee of the Ghana Health Services. Information on the ethical considerations of the DHS survey can be obtained online (www.dhsprogram.com). Nonetheless, permission was obtained from ICF to use the dataset. Moreover, the dataset was used for the sole purpose of this study and the sources from which relevant ideas were obtained for this study have been duly referenced.

## Results

### Sociodemographic characteristics of study participants

The ages of the children ranged from 0–59 months with a mean age of 28.36 ±17.10 months. Of the total children, 11.38% were less than 6 months old. The majority of children were males (52.12%), resident in rural settings (60.84%), from households with 5–9 members (57.59%), households with access to improved toilet facilities (58.22%) and improved drinking water sources (70.54%). About 33% of the children lived in the poorest households. The mean age of the mothers was 30.65±6.89 years. The majority of mothers delivered the index child in a health facility (67.94%), and 69.80% were registered unto the National Health Insurance Scheme (NHIS) (Table 1).

**Table 1:** Sociodemographic characteristics of study participants (N = 2636 unless specified).

Variable/category	Number	Percentage
**Age (months)**		
0–5	300	11.38
6–11	260	9.86
12–23	568	21.55
24–35	537	20.37
≥36	971	36.84
**Sex**		
Male	1,374	52.12
Female	1,262	47.88
**Immediate factors**		
**Birth weight (N = 1533)**		
<2.5 kg	160	10.44
≥2.5 kg	1,373	89.56
**Dietary diversity score (N = 1659)**		
Below minimum	1335	80.47
Minimum	324	19.53
**Birth order number of child**		
1^st^	554	21.02
2^nd^	556	21.09
3^rd^	453	17.19
4^th^	383	14.53
≥5^th^	690	26.17
**Fever & cough in the last 2 weeks**		
No	2475	93.89
Yes	161	6.11
**Diarrhoea in the last 2 weeks**		
No	2299	87.22
Yes	337	12.78
**Intermediate factors**		
**Mother's age (years)**		
15–19	96	3.64
20–29	1104	41.88
30–39	1119	42.45
40–49	317	12.03
**Parity**		
1	418	15.86
2	597	22.65
3	546	20.71
4	396	15.02
≥5	679	25.76
**Mother's BMI category (N = 2632)**		
Thin	143	5.43
Normal	1,570	59.65
Overweight	919	34.92
**Timing of first ANC visit (N = 1923)**		
First trimester	1,262	65.63
Second trimester and beyond	661	34.37
**Place of delivery**		
Home	845	32.06
Health facility	1,791	67.94
**Health insurance coverage**		
No	796	30.20
Yes	1,840	69.80
**Woman's autonomy tertiles**		
Low autonomy	1,001	37.99
Middle autonomy	1,011	38.34
High autonomy	624	23.67
**Household size**		
≤4	892	33.84
5–9	1,518	57.59
≥10	226	8.57
**Type of toilet facility (N = 2583)**		
Unimproved	1,079	41.78
Improved	1,504	58.22
**Source of drinking water (N = 2583)**		
Unimproved	761	29.46
Improved	1,822	70.54
**Distal factors**		
**Mother's educational level**		
No formal education	923	35.02
Primary	562	21.32
Secondary or higher	1,151	43.66
**Husband/partner's educational level (N = 2448)**		
No formal education	782	31.94
Primary	277	11.32
Secondary or higher	1,389	56.74
**Mother's employment status (N = 2633)**		
Unemployed	457	17.36
Employed	2176	82.64
**Religion**		
Traditional	241	9.14
Islam	539	20.45
Christian	1,856	70.41
**Region**		
Western	263	9.97
Central	258	9.79
Greater Accra	209	7.92
Volta	218	8.26
Eastern	253	9.6
Ashanti	236	8.95
Brong Ahafo	304	11.53
Northern	427	16.2
Upper East	242	9.2
Upper West	226	8.58
**Place of residence**		
Urban	1,032	39.16
Rural	1,604	60.84
**Wealth index**		
Poorest	864	32.77
Poorer	581	22.04
Middle	490	18.59
Richer	394	14.95
Richest	307	11.65

### Prevalence and distribution of underweight, wasting and stunting in children under five years in Ghana by age and sex

From Table 2, the prevalence of underweight, wasting and stunting was 10.43% [95% CI: 9.10–11.93], 5.31% [95% CI: 4.34–6.48] and 18.37% [95% CI: 16.60–20.28] respectively. There were significant differences between groups for all the three forms of undernutrition by child’s age category. The highest prevalence of underweight (12.81%), wasting (11.44%) and stunting (28.60%) was found among children aged 12–23, 6–11 and 24–35 months, respectively (p<0.05). Female children (16.44%) were less prone to stunting than their male counterparts (20.15%) (p = 0.033).

**Table 2:** Prevalence (%) and distribution of underweight, wasting and stunting in children under five years in Ghanaby age and sex (N = 2636 unless stated).

Variable	Underweight		Wasting		Stunting	
	%[95% CI]	P-value	%[95% CI]	P-value	%[95% CI]	P-value
**All children**	10.43[9.05–11.99]		5.31[4.36–6.44]		18.37[16.56–20.34]	
**Age (months)**		0.020		<0.001		<0.001
0–5	6.06[3.49–10.32]		9.82[6.57–14.43]		5.30[3.17–8.74]	
6–11	11.49[8.02–16.18]		11.44[7.31–17.46]		7.84[5.16–11.74]	
12–23	12.81[10.24–15.91]		7.42[5.23–10.44]		18.58[15.14–22.58]	
24–35	12.55[9.51–16.39]		3.52[2.09–5.89]		28.60[24.18–33.46]	
≥36	8.93[6.98–11.34]		2.02[1.07–3.77]		19.46[16.48–22.83]	
**Sex**		0.633		0.576		0.033
Male	10.73 [8.92–12.86]		5.02 [3.74–6.71]		20.15[17.74–22.79]	
Female	10.09 [8.27–12.26]		5.62 [4.32–7.27]		16.44[14.05–19.16]	

### Determinants of underweight, wasting and stunting in children under five years in Ghana

#### Determinants of underweight among children under five years in Ghana

Child’s age, birthweight and mother’s BMI category were significant determinants of underweight after controlling for confounding by the other factors. Underweight was more prevalent among children aged 12–23 months (AOR = 10.65, 95% CI: 1.79–63.32) and 24–35 months (AOR = 11.05, 95% CI: 1.89–64.69) compared to children aged 0–5 months. Relatively, low weight at birth was associated with a higher odds of underweight (AOR = 4.41, 95% CI: 2.17–8.97). Women in the normal and overweight BMI categories were less likely to have underweight children (AOR = 0.36, 95% CI: 0.14–0.92 and AOR = 0.29, 95% CI: 0.10–0.84, respectively) (Table 3).

**Table 3:** Determinants of underweight among children under five years in Ghana.

Variable/category	Model 1	Model 2	Model 3
	AOR[95% CI]	AOR[95% CI]	AOR[95% CI]
**Age (months)**			
0–5 (Ref)			
6–11	2.05[0.93–4.51]*	1.77[0.79–3.99]	7.87[1.14–54.31]***
12–23	2.48[1.22–5.06]***	2.17[1.00–4.70]*	10.65[1.79–63.32]***
24–35	2.56[1.26–5.17]***	2.10[0.93–4.77]*	11.05[1.89–64.69]***
≥36	1.65[0.83–3.28]	1.29[0.52–3.16]	6.12[1.00–37.53]
**Sex**			
Male (Ref)			
Female	0.89[0.66–1.20]	1.01[0.71–1.44]	0.84[0.48–1.48]
**Distal level factors**			
**Mother's educational level**			
No formal education (Ref)			
Primary	0.93[0.59–1.46]	0.60[0.35–1.03]	0.95[0.47–1.92]
Secondary or higher	0.62[0.37–1.03]*	0.44[0.25–0.76]*	0.53[0.24–1.16]
**Husband/partner's educational level**			
No formal education (Ref)			
Primary	1.72[0.99–2.98]*	1.94[1.07–3.51]***	2.18[0.92–5.17]
Secondary or higher	0.81[0.50–1.33]	1.09[0.64–1.87]	0.54[0.25–1.16]
**Mother's employment status**			
Unemployed (Ref)			
Employed	0.71[0.44–1.14]		
**Religion**			
Traditional (Ref)			
Islam	1.06[0.57–1.95]		
Christian	1.14[0.68–1.92]		
**Region**			
Western (Ref)			
Central	1.14[0.59–2.20]	1.26[0.55–2.86]	
Greater Accra	0.76[0.32–1.84]	0.71[0.22–2.24]	
Volta	0.63[0.31–1.27]	0.46[0.15–1.39]	
Eastern	0.69[0.30–1.56]	0.65[0.23–1.83]	
Ashanti	0.97[0.44–2.14]	0.78[0.27–2.22]	
Brong Ahafo	0.41[0.20–0.83]***	0.54[0.23–1.28]	
Northern	1.30[0.67–2.50]	1.04[0.44–2.34]	
Upper East	0.79[0.39–1.57]	0.95[0.39–2.34]	
Upper West	0.79[0.39–1.60]	0.74[0.29–1.89]	
**Place of residence**			
Urban (Ref)			
Rural	1.22[0.75–1.97]		
**Wealth index**			
Poorest (Ref)			
Poorer	1.02[0.65–1.59]	1.09[0.64–1.86]	
Middle	0.60[0.34–1.07]*	0.53[0.24–1.17]	
Richer	0.73[0.38–1.40]	1.22[0.58–2.56]	
Richest	0.37[0.16–0.88]***	0.64[0.24–1.73]	
**Intermediate factors**			
**Mother's age (years)**			
15–19 (Ref)			
20–29		0.50[0.17–1.42]	
30–39		0.54[0.16–1.76]	
40–49		0.76[0.19–3.03]	
**Parity**			
1 **(Ref)**			
2		0.57[0.30–1.08]*	
3		0.71[0.32–1.58]	
4		0.65[0.27–1.56]	
≥5		0.47[0.19–1.17]	
**Mother's BMI category (N = 2632)**			
Thin (Ref)			
Normal		0.53[0.27–1.22]	0.36[0.14–0.92]***
Overweight		0.23[0.11–0.50]**	0.29[0.10–0.84]***
**Timing of first ANC visit (N = 1923)**			
First trimester (Ref)			
Second trimester and beyond		1.21[0.82–1.79]	
**Place of delivery**			
Health facility (Ref)			
Home		0.92[0.57–1.49]	
**Health insurance coverage**			
Yes (Ref)			
No		1.19[0.78–1.80]	
**Woman's autonomy tertiles**			
Low autonomy (Ref)			
Middle autonomy		0.80[0.52–1.22]	
High autonomy		0.78[0.46–1.34]	
**Household size**			
≤4 (Ref)			
5–9		1.21[0.72–2.05]	
≥10		0.90[0.43–1.90]	
**Type of toilet facility (N = 2583)**			
Improved (Ref)			
Unimproved		1.06[0.62–1.81]	
**Source of drinking water (N = 2583)**			
Improved (Ref)			
Unimproved		0.94[0.61–1.42]	
**Immediate factors**			
**Birth weight (N = 1533)**			
≥2.5 kg (Ref)			
<2.5 kg			4.41[2.17–8.97]**
**Dietary diversity score (N = 1659)**			
Below minimum (Ref)			
Minimum			1.20[0.63–2.29]
**Birth order number of child**			
1^st^ (Ref)			
2^nd^			0.58[0.23–1.42]
3^rd^			0.94[0.40–2.23]
4^th^			0.87[0.32–2.39]
≥5th			1.14[0.53–2.46]
**Fever & cough in the last 2 weeks**			
No (Ref)			
Yes			0.46[0.16–1.31]
**Diarrhoea in the last 2 weeks**			
No (Ref)			
Yes			1.11[0.59–2.10]
**The goodness of fit test results**	F-adjusted test statistic = F = (9,386) = 0.970, P = 0.464	F-adjusted test statistic = F = (9,386) = 0.271, P = 0.982	F-adjusted test statistic = F = (9,314) = 1.317, P = 0.227

*p<0.1,

**p<0.001,

***p<0.05, Ref: Reference group

#### Determinants of wasting among children under five years in Ghana

The determinants of wasting among children under five years have been presented in Table 4. In the final model, child’s age, sex, dietary diversity score, husband/partner’s educational level, and wealth index were found to be significantly associated with wasting. Comparatively, wasting was less prevalent among children in the age groups of 24–35 months (AOR = 0.24, 95% CI: 0.07–0.79) and 36 months and over (AOR = 0.09, 95% CI: 0.02–0.43). Also, children from the middle-class of the household wealth index were less likely to be wasted (AOR = 0.31, 95% CI: 0.10–0.97). However, female sex (AOR = 2.52, 95% CI: 1.18–5.41), minimum DDS (AOR = 2.46, 95% CI: 1.12–5.39), fourth birth order number of child (AOR = 3.29, 95% CI:1.01–10.71), primary level husband/partner education (AOR = 4.12, 95% CI: 1.39–12.21) were associated with a higher odds of wasting.

**Table 4:** Determinants of wasting among children under five years in Ghana.

Variable/category	Model 1	Model 2	Model 3
	AOR[95% CI]	AOR[95% CI]	AOR[95% CI]
**Age (months)**			
0–5 (Ref)			
6–11	1.16[0.61–2.21]	0.78[0.38–1.59]	0.72[0.22–2.34]
12–23	0.71[0.37–1.35]	0.56[0.27–1.14]	0.56[0.20–1.57]
24–35	0.36[0.17–0.75]***	0.30[0.14–0.65]***	0.24[0.07–0.79]***
≥36	0.19[0.08–0.42]**	0.08[0.03–0.24]**	0.09[0.02–0.43]***
**Sex**			
Male (Ref)			
Female	1.10[0.71–1.71]	1.48[0.95–2.31]*	2.52[1.18–5.41]***
**Distal level factors**			
**Mother's educational level**			
No formal education (Ref)			
Primary	0.82[0.42–1.63]		
Secondary or higher	0.90[0.44–1.84]		
**Husband/partner's educational level**			
No formal education (Ref)			
Primary	1.93[0.94–3.96]*	2.15[1.03–4.51]***	4.12[1.39–12.21]***
Secondary or higher	1.28[0.65–2.50]	1.26[0.66–2.43]	2.43[1.02–5.77]
**Mother's employment status**			
Unemployed (Ref)			
Employed	1.03[0.62–1.72]		
**Religion**			
Traditional (Ref)			
Islam	2.31[1.06–5.02]***	2.69[1.10–6.63]***	1.68[0.34–8.29]
Christian	1.26[0.62–2.58]	1.30[0.52–3.24]	0.63[0.15–2.65]
**Region**			
Western (Ref)			
Central	1.26[0.49–3.28]		
Greater Accra	0.73[0.21–2.52]		
Volta	0.70[0.20–2.44]		
Eastern	0.94[0.28–3.12]		
Ashanti	1.03[0.33–3.21]		
Brong Ahafo	1.10[0.40–3.09]		
Northern	1.19[0.42–3.36]		
Upper East	1.79[0.66–4.88]		
Upper West	1.24[0.41–3.76]		
**Place of residence**			
Urban (Ref)			
Rural	1.56[0.83–2.94]		
**Wealth index**			
Poorest (Ref)			
Poorer	0.70[0.35–1.41]	0.71[0.34–1.47]	0.91[0.36–2.29]
Middle	0.40[0.16–0.97]***	0.31[0.11–0.81]***	0.31[0.10–0.97]***
Richer	0.90[0.33–2.46]	0.85[0.35–2.10]	0.40[0.13–1.26]
Richest	0.91[0.23–3.56]	0.67[0.21–2.14]	0.45[0.13–1.55]
**Intermediate factors**			
**Mother's age (years)**			
15–19 (Ref)			
20–29		1.34[0.30–6.03]	
30–39		1.30[0.27–6.38]	
40–49		2.51[0.38–16.61]	
**Parity**			
1 (Ref)			
2		0.58[0.28–1.19]	
3		0.85[0.35–2.04]	
4		0.99[0.39–2.51]	
≥5		0.40[0.15–1.12]*	
**Mother's BMI category (N = 2632)**			
Thin (Ref)			
Normal		1.21[0.45–3.21]	
Overweight		0.68[0.22–2.10]	
**Timing of first ANC visit (N = 1923)**			
First trimester (Ref)			
Second trimester and beyond		1.30[0.83–2.02]	
**Place of delivery**			
Health facility (Ref)			
Home		1.04[0.59–1.84]	
**Health insurance coverage**			
Yes (Ref)			
No		1.80[1.06–3.06]***	1.40[0.63–3.14]
**Woman's autonomy tertiles**			
Low autonomy (Ref)			
Middle autonomy		1.01[0.54–1.88]	
High autonomy		1.46[0.76–2.80]	
**Household size**			
≤4 (Ref)			
5–9		1.54[0.79–3.00]	
≥10		0.66[0.24–1.86]	
**Type of toilet facility (N = 2583)**			
Improved (Ref)			
Unimproved		1.33[0.74–2.39]	
**Source of drinking water (N = 2583)**			
Improved (Ref)			
Unimproved		0.87[0.49–1.55]	
**Immediate factors**			
**Birth weight (N = 1533)**			
≥2.5 kg (Ref)			
<2.5 kg			2.20[0.91–5.35]*
**Dietary diversity score (N = 1659)**			
Below minimum			
Minimum			2.46[1.12–5.39]***
**Birth order number of child**			
1^st^ (Ref)			
2^nd^			1.12[0.35–3.61]
3^rd^			0.65[0.18–2.28]
4^th^			3.29[1.01–10.71]***
≥5th			0.98[0.30–3.27]
**Fever & cough in the last 2 weeks**			
No (Ref)			
Yes			1.90[0.56–6.50]
**Diarrhoea in the last 2 weeks**			
No (Ref)			
Yes			1.56[0.62–3.94]
**The goodness of fit test results**	F-adjusted test statistic = F = (9,386) = 4.168, P<0.001	F-adjusted test statistic = F = (9,386) = 4.168, P = 0.007	F-adjusted test statistic = F = (9,315) = 0.337, P = 0.962

*p<0.1,

**p<0.001,

***p<0.05, Ref: Reference group

#### Determinants of stunting among children under five years in Ghana

Independently, child’s age, birth weight, the birth order of the child, woman’s autonomy and region were identified as determinants of stunting. Stunting was more prevalent among children aged 12–23 months (AOR = 4.54, 95% CI: 1.40–14.74), 24-35months (AOR = 9.99, 95% CI: 3.30–30.24) and 36 months and over (AOR = 5.13, 95% CI: 1.67–15.80). Relatively, children born low birth weight were 3 times more likely to be stunted (AOR = 3.18, 95% CI: 1.70–5.92). Also, birth order of 5 or more was associated with 2 times the odds of stunting (AOR = 2.03, 95% CI: 1.04–3.96). A high woman’s autonomy was associated with a lower odds stunting (AOR = 0.52, 95% CI: 0.28–0.97) whereas children from the northern region of Ghana were about 3 times (AOR = 2.99, 95% CI: 1.13–7.94) more likely to be stunted (Table 5).

**Table 5:** Determinants of stunting among children under five years in Ghana.

Variable/category	Model 1	Model 2	Model 3
	AOR[95% CI]	AOR[95% CI]	AOR[95% CI]
**Age (months)**			
0–5 (Ref)			
6–11	1.95[0.82–4.63]	1.21[0.52–2.82]	1.79[0.45–7.15]
12–23	5.82[2.85–11.88]**	3.32[1.76–6.25]**	4.54[1.40–14.74]***
24–35	10.17[5.12–20.20]**	6.68[3.46–12.52]**	9.99[3.30–30.24]**
≥36	5.81[3.02–11.17]**	4.18[2.18–8.01]**	5.13[1.67–15.80]***
**Sex**			
Male (Ref)			
Female	0.77[0.61–0.97]***	0.72[0.55–0.95]***	0.67[0.42–1.06]
**Distal level factors**			
**Mother's educational level**			
No formal education (Ref)			
Primary	1.04[0.73–1.48]	0.69[0.45–1.07]	1.18[0.65–2.16]
Secondary or higher	0.55[0.37–0.82]***	0.56[0.37–0.85]*	0.75[0.42–1.35]
**Husband/partner's educational level**			
No formal education (Ref)			
Primary	1.08[0.71–1.65]		
Secondary or higher	0.89[0.61–1.30]		
**Mother's employment status**			
Unemployed (Ref)			
Employed	0.69[0.47–1.01]*	1.19[0.77–1.83]	
**Religion**			
Traditional (Ref)			
Islam	0.51[0.30–0.87]***	0.82[0.41–1.65]	
Christian	0.69[0.41–1.18]	0.85[0.46–1.59]	
**Region**			
Western (Ref)			
Central	0.94[0.54–1.64]	1.08[0.57–2.06]	0.91[0.29–2.85]
Greater Accra	0.55[0.25–1.19]	0.59[0.26–1.31]	0.80[0.28–2.29]
Volta	0.54[0.29–1.00]*	0.45[0.21–0.98]***	1.32[0.42–4.08]
Eastern	0.73[0.41–1.30]	0.77[0.36–1.65]	1.09[0.35–3.37]
Ashanti	0.80[0.42–1.46]	0.62[0.28–1.36]	0.77[0.28–2.10]
Brong Ahafo	0.59[0.31–1.10]*	0.61[0.33–1.12]	0.84[0.32–2.18]
Northern	1.36[0.76–2.46]	1.16[0.57–2.34]	2.99[1.13–7.94]***
Upper East	0.62[0.34–1.14]	0.71[0.34–1.50]	0.66[0.21–2.05]
Upper West	0.84[0.47–1.50]	0.76[0.37–1.56]	1.32[0.48–3.63]
**Place of residence**			
Urban (Ref)			
Rural	0.93[0.63–1.36]		
**Wealth index**			
Poorest (Ref)			
Poorer	1.18[0.77–1.81]	1.47[0.91–2.40]	
Middle	0.83[0.51–1.35]	0.86[0.49–1.50]	
Richer	0.77[0.42–1.40]	1.15[0.60–2.21]	
Richest	0.37[0.17–0.81]***	0.54[0.23–1.24]	
**Intermediate factors**			
**Mother's age (years)**			
15–19 (Ref)			
20–29		0.53[0.26–1.10]*	
30–39		0.58[0.25–1.33]	
40–49		0.56[0.21–1.50]	
**Parity**			
1 (Ref)			
2		0.72[0.45–1.15]	
3		0.79[0.45–1.39]	
4		0.94[0.47–1.91]	
≥5		0.87[0.43–1.79]	
**Mother's BMI category (N = 2632)**			
Thin (Ref)			
Normal		0.72[0.39–1.32]	0.93[0.34–2.52]
Overweight		0.46[0.24–0.89]***	0.89[0.31–2.56]
**Timing of first ANC visit (N = 1923)**			
First trimester (Ref)			
Second trimester and beyond		1.30[0.95–1.79]	
**Place of delivery**			
Health facility (Ref)			
Home		0.70[0.48–1.02]*	0.93[0.41–2.13]
**Health insurance coverage**			
Yes (Ref)			
No		0.99[0.72–1.36]	
**Woman's autonomy tertiles**			
Low autonomy (Ref)			
Middle autonomy		1.01[0.73–1.39]	0.75[0.46–1.24]
High autonomy		0.61[0.40–0.91]***	0.52[0.28–0.97]***
**Household size**			
≤4 (Ref)			
5–9		0.91[0.60–1.39]	
≥10		0.73[0.37–1.43]	
**Type of toilet facility (N = 2583)**			
Improved (Ref)			
Unimproved		1.15[0.79–1.68]	
**Source of drinking water (N = 2583)**			
Improved (Ref)			
Unimproved		0.92[0.64–1.32]	
**Immediate factors**			
**Birth weight (N = 1533)**			
≥2.5 kg (Ref)			
<2.5 kg			3.18[1.70–5.92]**
**Dietary diversity score (N = 1659)**			
Below minimum (Ref)			
Minimum			1.02[0.59–1.74]
**Birth order number of child**			
1^st^ (Ref)			
2^nd^			0.95[0.49–1.84]
3^rd^			1.28[0.61–2.67]
4^th^			1.30[0.62–2.72]
≥5th			2.03[1.04–3.96]***
**Fever & cough in the last 2 weeks**			
No (Ref)			
Yes			0.82[0.33–2.02]
**Diarrhoea in the last 2 weeks**			
No (Ref)			
Yes			0.68[0.35–1.33]
**The goodness of fit test results**	F-adjusted test statistic = F = (9,386) = 0.979, P = 0.457	F-adjusted test statistic = F = (9,390) = 1.140, P = 0.333	F-adjusted test statistic = F = (9,326) = 1.636, P = 0.104

*p<0.1,

**p<0.001,

***p<0.05, Ref: Reference group

### Robustness checks of results

The robustness of our results was ensured and checked. The “svy” command prefix was used in all analyses to adjust for the study design used by the DHS program. The Taylor series linearization method was used to estimate confidence intervals. Survey logistic regressions were used to identify the determinants of undernutrition to account for the study design. The fit of our final models was assessed using the “svylogitgof” which is used for the estimation of the goodness of fit after survey estimations [46]. We found no evidence of lack of fit of any of our final models as the *P*-values produced were greater than 0.05.

## Discussion

This study sought to estimate the prevalence of underweight, wasting and stunting among children under five years and identify the determinants of undernutrition in Ghana. The findings showed that the prevalence of underweight, wasting and stunting was 10.4%, 5.3%, and 18.4% respectively. These rates of undernutrition are lower than the rates reported from other countries in Africa, such as Niger, Burundi, Ethiopia, and Mozambique [47,48]. Over the past decade, the Government of Ghana and the Ministry of Health (MoH) have taken concentrate steps in improving maternal, child health and nutrition through the implementation of free antenatal care services, iron and folate supplementation for pregnant women and school feeding activities. These strategies may have contributed to the reduction in the prevalence of undernutrition in children under five years in Ghana. Comparatively, food crises, limited access to arable land for agriculture purposes and adverse climatic conditions have constrained progress in tackling undernutrition in Niger, Burundi, Ethiopia and Mozambique [47,49]; accounting partly for the differences in the prevalence rates of undernutrition between Ghana and the other countries.

The findings also revealed that a child’s age is a determinant of underweight, wasting and stunting. Whereas, the sex is a determinant of wasting and stunting. The risk of underweight increased from the age of 6 months and may be related to the fact that most children in Ghana are breastfed in early life until at 6 months when they transition from only breastfeeding to feeding family foods in addition to breastfeeding which in most cases is characterized by challenges [50,51]. Furthermore, as the age increased from 24 months, the risk of wasting is reduced. On the other hand, stunting expressed itself in children after 11 months but peaked around 24–35 months old. Relatively, male children were more likely to be stunted before the age of 3 years. However, female children were more likely to be wasted than their male counterparts. These findings have been corroborated by other studies [19,31,32,51,52]. The high levels of stunting after 11 months may be explained by repeated exposure to nutritional insults from the prenatal period (first 1000 days).

Nevertheless, the positive association between male sex and stunting has remained controversial. A meta-analysis on gender and stunting concluded that boys in sub-Saharan Africa were more likely to be stunted in early childhood than girls [53]; this could be due to differences in behaviours, gender inequalities and the biological susceptibility of males to morbidity in early infancy [54]. However, wasting results from inadequate intake of quality food, and it has been reported elsewhere that in food insecure households, undernutrition is more likely to show in female children relative to males [55].

At the immediate level, low birth weight was associated with an increased risk of underweight and stunting, which supports findings from a study in Botswana [6]. A child born low birth weight has already suffered from intrauterine growth retardations and is undernourished at the time of birth [55]. This undernourished infant may live to be undernourished in early childhood even in the presence of favourable conditions as these conditions may not be sufficient enough to fully compensate for the initial damage caused to the child at birth [29]. Surprisingly, children who met the minimum DDS were more likely to be wasted compared to their counterparts. This finding contradicts earlier studies in Ghana that reported an inverse relationship between adherence to the minimum DDS and undernutrition [27,56]. The DDS assesses the quality of the child’s food and was assessed with a qualitative 24-hour dietary recall in the DHS. Based on this, the results may not necessarily reflect the usual dietary pattern of the child. Additionally, the DDS was liable to changes in household living conditions as well as the effects of seasonality on food. It is established that seasonal variations can affect the availability and access to especially vitamin A and C-rich foods and in turn, affect the quality and quantity of food consumed by households [57]. More importantly, the design of the study was cross-sectional, and it may be that undernourished children received extra care in the quality of their food thus, explaining the inverse association between MDDS and undernutrition in this study.

Women are often regarded as primary caregivers; therefore, high woman’s autonomy defined by her freedom to take independent decisions, participate in key decision-making processes and have more control over household resources can impact significantly on decision making with regards to her nutrition and the nutrition of the children [47,58]. This explains the positive association between high autonomy and reduced odds of childhood undernutrition in this study. Also, we found a negative association between maternal BMI category and child underweight. Maternal BMI influences the child’s nutritional status, pre-and-post pregnancy period. During pregnancy, the fetus in an undernourished woman (thin BMI category) is predisposed to adverse growth shocks that contribute to intrauterine growth restrictions [1]. Also, an undernourished woman faces challenges that include difficulty with breastfeeding, reduced mental abilities, and energy levels that can hinder her ability to take proper care of her children [59]. The association between maternal undernutrition and poor nutritional outcomes has been reported in Rwanda [8]. This study did not find any significant association between household size and undernutrition. However, a higher birth order number of the child was significantly associated with an increased odds of wasting and stunting; which could be due to adverse conditions either after delivery or during pregnancy that may have resulted from multiple births by the woman.

At the distal level, children from middle-class households were less likely to be stunted whereas primary level paternal education and residing in the northern region of Ghana were associated with an increased risk of wasting and stunting respectively. Undernutrition is a direct representation of poverty. Individuals from poor households are predisposed to poor living conditions that may result in diseases and poor health outcomes [32]. Comparatively, middle-class households are financially capable, food secured and have better access to health care services, which are precursors for optimal childhood nutritional outcomes [59]. The association between increased wealth index and low rates of undernutrition has been reported by other studies [8,10]. A husband/partner’s influence on a child’s nutritional status can be mediated through other factors and not directly. A husband with low educational status (less than secondary) may not be gainfully employed, resulting in poor household wealth status, food insecurity, and poor general living conditions. Additionally, males are more involved in child care by providing financial and physical support to the woman and are less involved in decision making regarding appropriate infant and young child feeding practices [60]. The northern region of Ghana lies in the savannah zone. Hence, the high prevalence of stunting in the region can partly be explained by the ecological constraints, food insecurity and poverty in the region [32,52].

Some strengths and limitations should be taken into account when interpreting the findings of this study. The study participants in the GDHS 2014 were women who delivered in the past five years preceding the survey. The extended period, therefore, subjects some parts of the data such as the timing of the first ANC visits to recall biases except for the anthropometric measurements. The magnitude of the recall bias is unknown and correcting it is impossible. However, it is assumed that the biases are to a moderately sufficient extent random. The DHS survey also employed a cross-sectional study design and therefore, does not depict any causal link. We, therefore, limit our interpretation to describing associations. Nonetheless, we thoroughly modelled several potential explanatory variables including subject and household demographics, education, livelihood and socio-economic conditions, which may be contributing to child nutritional status. Lastly, we employed robust statistical models with weighting factors while accounting for the effects of clustering and stratification in the data.

## Conclusion

The aetiology of undernutrition in Ghana is multifaceted and interconnected. The nutritional status of children under five years is determined by socioeconomic, cultural, household, maternal, and child level factors. The effect of socioeconomic factors on underweight, wasting and stunting is exhibited independently and mediated through maternal and child level factors.

It is logical to conclude that empowering women as well as improving the socioeconomic status of households may contribute significantly to reducing morbidity and mortality from undernutrition. We recommend that nutritional interventions to fight childhood undernutrition should take into consideration policies or strategies that would empower women, and address socioeconomic inequalities at the community level.

## Supporting information

S1 Table Woman’s autonomy score index. (DOCX)
