# Supplementary material for: The epidemiology of undernutrition and its determinants in children under five years in Ghana
Source: PLoS One. 2019 Jul 31;14(7):e0219665. doi: 10.1371/journal.pone.0219665 (PMC6668784; doi:10.1371/journal.pone.0219665)
Supplement: S1 Table — (DOCX) [file pone.0219665.s001.docx]

**S1 Table: woman’s autonomy and self esteem index**

| **Questions** | **Possible responses** | **Score** |
| --- | --- | --- |
| 1. **Household decision making** |  |  |
| ***Who has final say on :*** |  |  |
| 1. *Person who decides how respondent money is used* | *Respondent alone* | *1* |
| 1. *Respondent’s health care* | *Respondent and husband* | *1* |
| 1. *Making large household purchase* | *Respondent and other person* | *1* |
| 1. *Visits to family or relatives* | *Husband/partner alone* | *0* |
| 1. *What to do with money wif’s) earnings* | *Someone else* | *0* |
|  | *Other* | *0* |
| *Total score for household decision making (A) = score(a)+score(b)+score(c)+score(d)+score(e)*  *Maximum attainable score for final decision making =5* | | |
| 1. **Attitude toward wife beating** |  |  |
| ***Wife beating is justified if wife:*** |  |  |
| 1. *Goes out without telling husband* | *Yes* | 0 |
| 1. *Neglects the children* | *No* | 1 |
| 1. *Argues with husband* | *Don’t know* | - |
| 1. *Refuses to have sex with husband* |  |  |
| 1. *Burns the food* |  |  |
| *Total score for attitude towards wife beating (B)= score(a)+score(b)+score(c)+score(d)+score(e)*  *Maximum attainable score for wife beating =5* | | |
| 1. ***Property ownership:*** |  |  |
| ***Woman:*** |  |  |
| 1. *Owns a house alone or jointly* | *Does not own* | 0 |
| 1. *Owns land alone or jointly* | *Alone only* | 1 |
|  | *Jointly only* | 1 |
|  | *Both alone and jointly* | 1 |
| *Total score for property ownership (C) = score(a)+score(b)*  *Maximum attainable score for property ownership=2* | | |
| Overall score for woman’s autonomy= A+B+C  Maximum attainable score for woman’s autonomy =12 | | |
